# An insight into genes responsible for fosfomycin resistance among uropathogens of asymptomatic bacteriuria during pregnancy: A North Indian study

**DOI:** 10.1099/acmi.0.000623.v5

**Published:** 2023-12-11

**Authors:** Sajda Khatoon, Asfia Sultan, Fatima Khan, Tamkin Khan, Anuradha Singh

**Affiliations:** ^1^​ Department of Microbiology, Jawaharlal Nehru Medical College and Hospital, AMU, Aligarh, India; ^2^​ Department of Obstetrics and Gynaecology, Jawaharlal Nehru Medical College and Hospital, AMU, Aligarh, India

**Keywords:** agar dilution, antimicrobial resistance, asymptomatic bacteriuria, fosfomycin, pregnancy

## Abstract

**Purpose.:**

Asymptomatic bacteriuria (ASB) is a common finding during pregnancy. Effective antibiotic treatment could reduce its adverse effects on both mother and fetus. However, emerging antimicrobial resistance limits the treatment options. Fosfomycin might be a promising drug in this regard, as its resistance is still low. The aim of the study was to determine the antimicrobial susceptibility pattern of fosfomycin in isolates causing ASB by disc diffusion and agar dilution (in selected isolates), determine minimum inhibitory contribution (MIC) by agar dilution in isolates resistant by disc diffusion and detect the genes responsible for fosfomycin resistance.

**Methods.:**

This was a 2-year study carried in the Department of Microbiology, Jawaharlal Nehru Medical College and Hospital (JNMCH), Aligarh Muslim University (AMU), Aligarh. A total of 10 252 urine samples from asymptomatic pregnant females (18–45 years) attending the antenatal care (ANC) outpatient department (OPD) were submitted. Identification of pathogen and antimicrobial susceptibility testing (AST) was carried out as per standard methods of CLSI. There was phenotypic detection of methicillin-resistant *Staphylococcus aureus* (MRSA) and other *Staphylococcus species* (MRSS), high-level aminoglycoside resistance (HLAR), vancomycin resistant *Enterococci* (VRE) and *S. aureus* (VRSA), extended spectrum β-lactamase (ESBL) and carbapenem-resistant Enterobacterales (CRE). All the fosfomycin-resistant isolates (by disk diffusion) were tested by agar dilution. Conventional PCR was performed for *murA*, *fosA*, *uhpT* and *glpT* genes on all resistant isolates.

**Result.:**

In this study, the prevalence of ASB among pregnant females was 1173(11.4 %), in which *Escherichia coli* 495(42 %) was the predominant organism. The overall sensitivity of fosfomycin among Gram-positive cocci (GPC) and Gram-negative bacilli (GNB) was 99 % and 97.6 %, respectively. MRSA and MRSS accounted for 50 (66.6 %) and 71 (76 %), respectively. The highest rates of MIC >2048 µg ml^−1^ were shown by most isolates (mainly *E. coli *) on agar dilution. PCR studies revealed four *E. coli* strains possessed both *murA* (also present in one *K. pneumoniae* strain) and *glpT* genes. While only one isolate (* E. faecalis *) was positive for *fosA* gene. But none of the strain possessed the *uhpT* gene.

**Conclusion.:**

According to this study, *murA* and *glpT* genes were more frequent than *fosA*. We cannot comment on the prevalence and regional distribution of fosfomycin-resistant genes based on this preliminary study. Therefore, more Indian studies should be carried out to create awareness about the presence of genes in a particular area.

## Data Summary

No supporting external data is generated for this work. The authors provided all supporting data and protocols within the article.

## Introduction

The hormonal and physiological changes that occur during pregnancy predisposes a pregnant woman to asymptomatic bacteriuria (ASB) and its failure to treatment can have a harmful effect on both the mother and fetus [1]. It can cause pyelonephritis, as well as pre-eclampsia, preterm delivery and low-birth weight in infants [2]. Therefore, it is imperative to screen and treat ASB in every woman during pregnancy [3]. Various bacterial pathogens are involved in the aetiology, *Escherichia coli* being the most frequent in all forms of urinary tract infections (UTIs). Other bacteria commonly isolated are *Klebsiella species*, other members of Enterobacterales, *Staphylococcus saprophyticus, S. aureus* and *Enterococci* [4]. Despite the availability of a variety of therapeutic alternatives for the treatment of UTIs, clinicians frequently have difficulty in selecting the most appropriate antibiotic therapy due to the rapid evolution of multidrug resistant bacteria (MDR) [5]. The use of antimicrobials is further limited by extended spectrum β-lactamases (ESBL), AmpC production by Gram negative (GN) bacteria, and methicillin or vancomycin resistance in Gram-positive (GP) bacteria [6].

Fosfomycin trometamol is a suitable therapeutic option for uncomplicated UTIs since it has the benefits of single dosing, less side effects, no effect on the commensals of gastrointestinal system and being safe during pregnancy [7]. It acts by inhibiting the bacterial cell-wall synthesis by inactivating the enzyme UDP-N-acetylglucosamine-3-enol-pyruvyltransferase (MurA) [8]. It is found to be effective against several uropathogens including *E. coli *, *Citrobacter sp.*, *Enterobacter sp.*, *Klebsiella sp.*, *Serratia sp.* and *Enterococcus faecalis* [8]. However, due to the emergence of antimicrobial resistance, this drug also reported resistance, which could be due to multiple mechanisms, including chromosomally mediated mutations in the target (*murA*) or transporter (*glpT* and *uhpT*) genes or less frequently to plasmid-mediated genes (*fosA, fosB, fosC*) encoding glutathione S-transferases that renders the drug inactive [9]. Even though there were many similar works done internationally [10, 11]. But, to the best of our knowledge, this was the first Indian study exploring genes responsible for fosfomycin resistance. Previous Indian studies discussed only the phenotypic resistance of fosfomycin [12, 13].

This study aimed at determining the sensitivity patterns of pathogens causing ASB during pregnancy against other routine antibiotics and fosfomycin by disc diffusion and agar dilution (in selected isolates), to assess the MICs by agar dilution in isolates resistant by disc diffusion and to determine the genes responsible for fosfomycin resistance by conventional PCR.

## Methods

### Specimen collection

The research study was a prospective observational study, carried out in the Department of Microbiology, Jawaharlal Nehru Medical College and Hospital (JNMCH), Aligarh Muslim University (AMU), Aligarh, during a 2 year period (November 2019 to November 2021). The Clinical Microbiology Laboratory of JNMCH, Aligarh received 10 252 early morning, freshly voided, middle stream urine samples from asymptomatic pregnant women aged 18–45 years who attended the antenatal care (ANC), outpatient department (OPD) at the Department of Obstetrics and Gynaecology. The sample size was calculated using the formula 4pq/L [2], where prevalence (p) of ASB in pregnant females was taken as 5 % as per a study [14] by North *et al* in 1990, error (L) was taken as 5 %.

### Inclusion criteria

- Pregnant women without the symptoms of UTI.

### Exclusion criteria

- Pregnant women with symptoms of UTI (fever, lower abdominal pain, dysuria, frequency, urgency or burning micturition etc.)
- High-risk pregnancy (like Gestational Diabetes Mellitus or pre-eclampsia)
- History of intake of antibiotics prior to sample collection

The processing was done on Cysteine Lactose Electrolyte Deficient (CLED) agar plates by the semi-quantitative culture of urine using a calibrated loop [15]. According to Infectious Disease Society of America (IDSA) guidelines, 1 or more bacterial species in urine at predefined quantitative counts (≥10^5^ c.f.u. ml^–1^), regardless of pyuria, without the signs or symptoms of UTI is regarded as ASB [4]. Identification of pathogens were carried out by standard biochemical methods [16].

### Antibiotic susceptibility testing (AST)

Bacterial suspension of turbidity 0.5 McFarland was prepared from overnight cultures in normal saline (0.9 % NaCl solution), and cultured uniformly on Muller–Hinton agar. Following the guidelines [17] of Clinical and Laboratory Standards Institute (M100-ed 31), the zone of inhibition diameter (Table 1a, b and c) was measured after aerobic incubation of 18–24 h at 37 °C.

**Table 1.** List of antibiotics used for various uropathogens

**(a) Enterobacterales and nil fermenters (except** ***Pseudomonas species*** **)**							
**First line**	**Zone size**			**Second line**	**Zone size**		
	S	I	R		S	I	R
Amoxycillin clavulanic acid (20/10 µg)	≥18	14–17	≤13	Colistin (10 µg)	–	–	–
Cefixime (5 µg)	≥19	16–18	15	Piperacillin-tazobactam (100/10 µg)	≥21	18–20	≤ 17
Ceftriaxone (30 µg)	≥23	20–22	≤19	Minocycline (30 µg)	≥16	13–15	≤ 12
Amikacin (30 µg)	≥17	15–16	≤14				
Meropenem (10 µg)	≥23	20–22	≤19				
Cotrimoxazole (1.25/23.75 µg)	≥16	11–15	≤10				
Nitrofurantoin (300 µg)	≥17	15–16	≤14				
Norfloxacin (10 µg)	≥17	13–16	≤12				
Fosfomycin (200 µg) *	≥16	13–15	≤12				
**(b)** ***Pseudomonas species***							
**First line**	**Zone size**			**Second line**	**Zone size**		
	S	I	R		S	I	R
Ceftazidime (10 µg)	≥18	15–17	≤14	Colistin (10 µg)	–	–	–
Piperacillin-tazobactam (100/10 µg)	≥21	15–20	≤14				
Meropenem (10 µg)	≥19	16–18	≤15				
Gentamicin (30 µg)	≥15	13–14	≤12				
Levofloxacin (5 µg)	≥22	15–21	≤14				
Aztreonam (30 µg)	≥22	16–21	≤15				
Cefepime (30 µg)	≥18	15–17	≤14				
Nitrofurantoin (300 µg)	≥17	15–16	≤14				
**(c) Gram‑positive isolates**							
**First line**	**Zone size**						
	S	I	R				
Cefoxitin (30 µg)	≥22 (* S. aureus *) ≥25 (Other *Staph. sp.*)	–	≤ 21 (* S. aureus *) ≤ 24 (Other *Staph. sp*.)				
Cotrimoxazole (1.25/23.75 µg)	≥16	11–15	≤10				
Vancomycin (30 µg)	–	–	–				
Nitrofurantoin (300 µg)	≥17	15–16	≤14				
Fosfomycin (200 µg) *	≥16	13–15	≤12				
Amikacin (30 µg)	≥17	15–16	≤14				
Norfloxacin (10 µg)	≥17	13–16	≤12				
Doxycycline (30 µg)	≥16	13–15	≤12				
Amoxiclav (20/10 µg)	≥18	14–17	≤13				
Ampicillin (10 µg)	≥17	–	≤16				
High-content gentamicin (120 µg)	≥10	–	≤16				
High-content streptomycin (300 µg)	≥10	–	≤6				

*The 200-µg fosfomycin disk contains 50 µg of glucose-6-phosphate.

*E. coli* ATCC 25922, *P. aeruginosa* ATCC 27853, *E. faecalis* ATCC 29212 and *S. aureus* ATCC 25923 were the strains used for quality control.

Vitek-2 system was further employed for identification of bacterial species and AST. Some phenotypic tests were employed for determination (Table 2) of ESBL, carbapenem-resistant Enterobacterales (CRE) and *Pseudomonas aeruginosa,* methicillin-resistant *S. aureus* (MRSA) and Other *Staph. sp.* (MRSS), high-level aminoglycoside resistance (HLAR) in *Enterococci* and vancomycin-resistant *S. aureus* (VRSA) and *Enterococci* (VRE).

**Table 2.** Phenotypic tests

Phenotypic detection of	Method	Result	QC
**ESBL**	By combination disc test method using ceftazidime (30 µg) versus ceftazidime- clavulanic acid (30/10 µg) on MHA, incubated at 35±2 °C for 16–18 h	≥5 mm increase in zone diameter of ceftazidime clavulanate vs ceftazidime alone=ESBL	* E. coli* ATCC 25922
**CRE and Carbapenem-resistant *P. aeruginosa ***	Some Enterobacterales produce carbapenemases	Shows resistance to one or more carbapenems (e.g. Imipenem, Meropenem etc.)	* E. coli* ATCC 25922
**MRSA and MRSS**	Cefoxitin disc diffusion test was performed on all *Staphylococcal* isolates using a 30 µg disc on MHA, incubated at 35°C for 18–24	Methicillin-resistant *S. aureus *: ≤21 mm Other *Staph. sp.*: ≤24 mm	* S. aureus* ATCC 25923
**HLAR in *Enterococcus* species**	Discs containing gentamycin (120 µg) and streptomycin (300 µg) were used on MHA, incubated at 35±2 °C in ambient air for 16–18 h	Gentamicin HLAR: 6 mm=resistant 7–9 mm=inconclusive ≥10 mm = susceptible	* E. faecalis* ATCC 29212 – susceptible * E. faecalis* ATCC 51299 – resistant
**VRSA and VRE by Vancomycin Screen Agar**	Agar dilution using BHI agar incorporating 6 µg ml^−1^ of vancomycin, incubated at 35±2 °C in ambient air for 24 h	VRSA: >1 colony=presumptive reduced susceptibility to vancomycin VRE: >1 colony=presumptive vancomycin resistance	* E. faecalis* ATCC 29212 – susceptible * E. faecalis* ATCC 51299 – resistant

### MIC determination of fosfomycin by agar dilution method

According to CLSI, AST of fosfomycin by disc diffusion and MIC breakpoints are solely applicable only to *E. coli* and *E. faecalis* urinary isolates [17]. Therefore, the accurate MIC testing method for organisms other than these is agar dilution (with 25 µg ml^−1^ glucose 6 phosphate enriched agar media) [17, 18]. Therefore, all fosfomycin-resistant isolates were confirmed by agar dilution (including random testing of fosfomycin sensitive isolates, 1 in 5) using doubling dilutions of fosfomycin (64, 128, 256, 512, 1024 and 2048 µg ml^−1^) with agar media containing 25 µg ml^−1^ of glucose 6 phosphate.

Colony suspension was made from an overnight incubated plate to obtain 0.5 McFarland turbidity. Using a micropipette, a 10 µl drop was spotted onto agar surface and then incubated aerobically at 35±2 °C for 24 h. The lowest concentration that inhibited the growth of bacteria was defined as the MIC. *E. coli* ATCC 25922 was used as a positive control.

The MIC of ≤64 µg ml^−1^ was considered sensitive, 128 g ml^−1^ as intermediate and ≥256 g ml^−1^ as resistant [17].

### PCR was performed for *murA*, *fosA*, *uhpT* and *glpT* genes

Conventional PCR was performed on all fosfomycin resistant isolates for the *murA*, *fosA*, *glpT* and *uhpT* genes (Table 3). We looked only for these four genes pertaining to constant availability of resources.

**Table 3.** List of primers used in the study

Genes	Primer	Primer sequences	Cycling conditions	Size	References
***murA***	Forward	ACGTAATGGTTCTGTGCATATTG-3	Initial denaturation: 94 °C for 5 min No. of cycles: 35 Denaturation: 94 °C for 1 min Annealing: 62 °C for 1 min Elongating: 72 °C for 1 min Extension: 72 °C for 7 min	984 bp	[10]
	Reverse	5-TAAATACGATCAACTACCGTCGT-3			
***fosA***	Forward	5-ATCTGTGGGTCTGCCTGTCGT-3	Initial denaturation: 95 °C for 5 min No. of cycles: 38 Denaturation: 94 °C for 40 s Annealing: 63.3 °C for 1 min Elongating: 72 °C for 1 min Extension: 72 °C for 7 min	271 bp	[34]
	Reverse	ATGCCCGCATAGGGCTTCT-3			
***glpT***	Forward	5′-GCGAGTCGCGAGTTTTCATTG-3′	Initial denaturation: 94 °C for 2 min No. of cycles: 35 Denaturation: 94 °C for 30 s Annealing: 55 °C for 30 s and Extension: 72 °C for 2 min Final extension: 72 °C for 5 min	1785 bp	[11]
	Reverse	5′-GGCAAATATCCACTGGCACC-3′			
***uhpT***	Forward	5′-TTTTTGAACGCCCAGACACC-3′	Same as *glpT*	1667 bp	[11]
	Reverse	5′-AGTCAGGGGCTATTTGATGG-3′			

Template DNA was prepared from freshly cultured bacterial strains by suspending two or three discrete bacterial colonies in 100 µl of molecular biology grade water, and then heating at 95 °C for 5 min and immediately chilling at 4 °C.

PCR assay was performed in 25 µl reaction volumes, which included 12.5 µl of Master Mix, 2.5 µl of Template DNA, 0.05 µl each of Forward and Reverse Primers, and 9.9 µl of Deionized Nuclease-free water. PCR mixture without template DNA was used as negative control. The gel electrophoresis was carried on 1.5 % (w/v) agarose (Bangalore Genei, India) in 10×TAE buffer (400 mM Tris, 200 mM acetic acid, 10 mM EDTA, pH 8.0), stained with dye ethidium bromide and visualized under UV light using a Gel Doc (Bio-Rad). Then 100 bp DNA ladder was used for *murA* and *fosA* genes whereas 1 kb plus DNA ladder (Invitrogen) for *uhpT* and *glpT* genes.

## Results

**Fig. 1.**
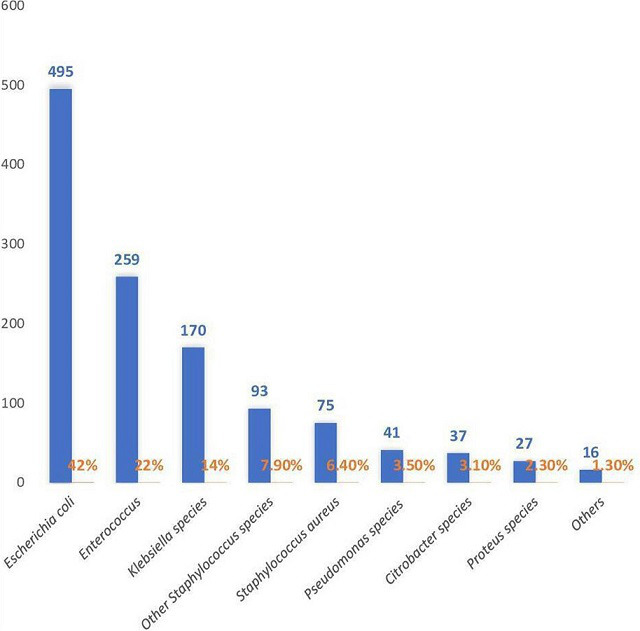
Prevalence of different pathogens of urinary tract responsible for asymptomatic bacteriuria during pregnancy (*n*=1173).

Of the total urine samples from females attending Obstetrics and Gynaecology OPD, 1526(14.6 %) were culture positive. Females having UTI symptoms accounted for 353(3.4 %) of the total, and they were excluded from the study. In total, 1173(11.4 %) pregnant females were found to have ASB (without the symptoms of UTI, with positive urine cultures). Altogether, 775(64 %) of the isolates were Gram-negative bacilli, with 731(62.3 %) comprised the order Enterobacterales. However, the frequency of Gram-positive pathogens was 438(36 %). *E. coli* 495(42 %) being the predominant organism (Fig. 1), followed by *Enterococci* 259(22 %). ‘Others’ included rare pathogens; *Streptococcus sp.* 11(0.9 %), *Acinetobacter sp.* 2(0.17 %)*, Serratia sp.* 2(0.17 %) and *Stenotrophomonas maltophilia* 1(0.08 %). Some cultures 40(3.4 %) showed mixed infection by two organisms.

**Fig. 2.**
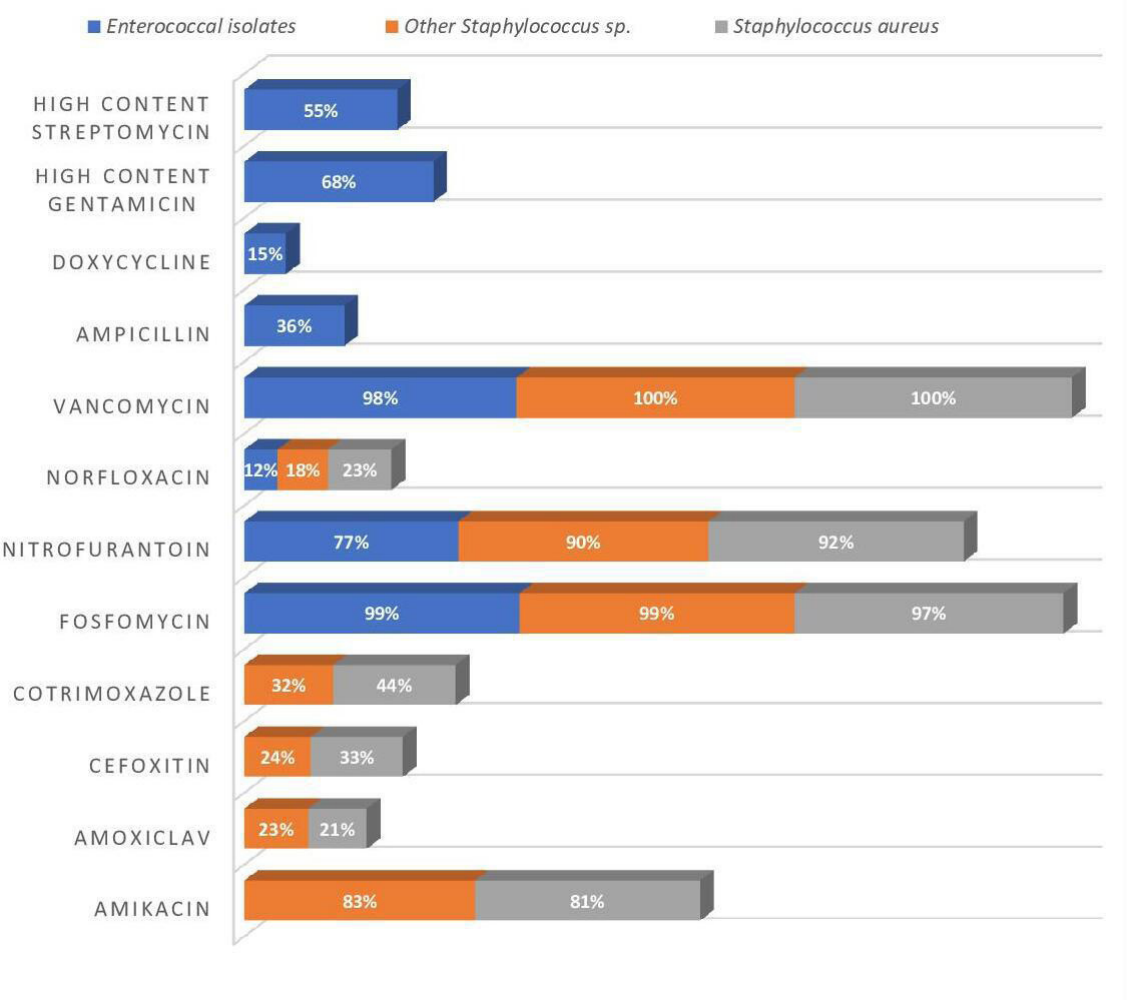
Antibiotic susceptibility pattern of Gram-positive urinary isolates (*n*=438) responsible for asymptomatic bacteriuria during pregnancy.

The overall susceptibility (Fig. 2) of vancomycin (99 %) was highest among Gram-positive bacteria, followed by aminoglycosides (83 %) and nitrofurantoin (82 %). Whereas flouroquinolones (FQs) (85 %) had high levels of resistance (Table S1, available in the online version of this article). MRSA as well as other MRSS accounted for 50(66.6 %) and 71(76 %), respectively. HLAR was found in 84(32 %) of the *Enterococcal* isolates. None of the isolates showed vancomycin resistance (VRSA/VRE) after routine testing by vancomycin screen agar.

**Fig. 3.**
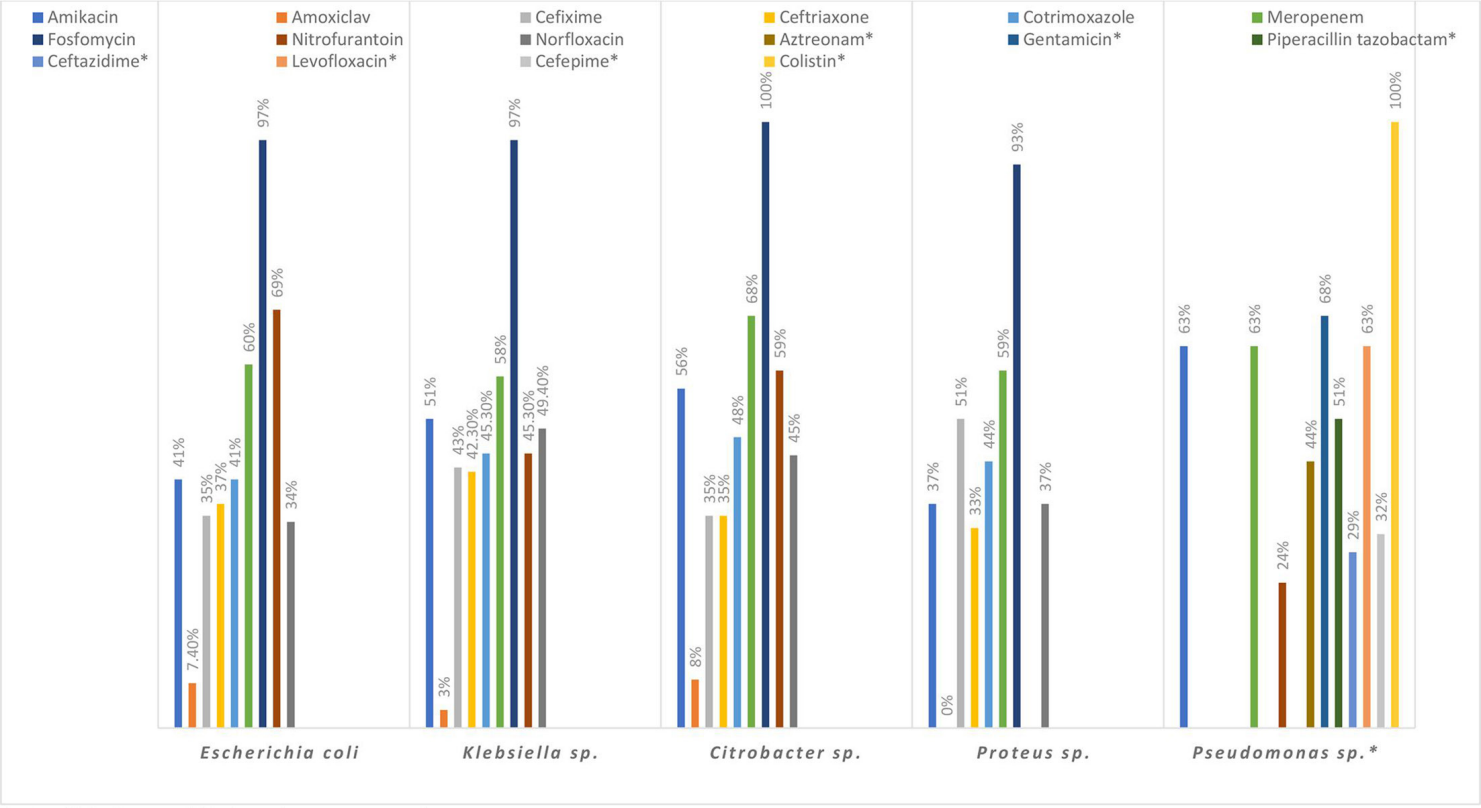
Antibiotic susceptibility pattern of Gram-negative urinary isolates (*n*=775) responsible for asymptomatic bacteriuria during pregnancy. *- Antibiotics used in *Pseudomonas* panel.

Among Gram-negative bacilli (Fig. 3), β-lactam (94 %) exhibited the highest resistance followed by FQs (62 %) and cephalosporins (62 %). The overall resistance ofcarbapenem among Enterobacteralesand *Pseudomonas* isolates were (40 %) and (36 %) respectively (Table S2).

The sensitivities of some rare pathogens (*n*=5), which include *Acinetobacter sp.* (*n*=2), *Serratia sp.* (*n*=2) and *Stenotrophomonas maltophilia* (*n*=1) was not depicted in Fig. 3/Table S2. None of them were resistant to fosfomycin, while only one isolate was susceptible to both nitrofurantoin and meropenem.

Fosfomycin showed excellent sensitivity of (99 %) and 97.6 % among GPCs and GNB, respectively. Fosfomycin was sensitive in all MRSA isolates and the susceptibility of fosfomycin in carbapenemase producing isolates and HLAR was extremely high 97 and 96%, respectively.

### Fosfomycin resistance

Isolates sensitive by disc diffusion were also sensitive (MICs<64 µg ml^−1^) by agar dilution. Total 22 fosfomycin-resistant isolates detected by disc diffusion, on further testing by agar dilution revealed one sensitive isolate (MIC <64 µg ml^−1^). So, we are left with 21 resistant isolates, among these 16 were GNR and 5 GPCs. Of these, *E. coli* contributed to 10 followed by four *Klebsiella sp.* and two *Proteus mirabilis *. Among the GPCs, there were two *E. faecalis* and two *S *. *aureus *, followed by one other *Staph. sp.* Among the Gram negatives, 9 (56 %) of them were ESBL producers.

On agar dilution, the majority of the resistant strains were *E. coli* with MIC >2048 µg ml^−1^ (Table 4).

**Table 4.** MIC determination of fosfomycin by agar dilution method

MICs	64 µg ml^−1^	128 µg ml^−1^	256 µg ml^−1^	512 µg ml^−1^	1024 µg ml^−1^	>2048 µg ml^−1^
**No. of Isolates**	1 (4.5 %)	3 (13.6 %)	3 (13.6 %)	None	2 (9 %)	13 (59 %)
**Bacterial species**	1 *E. coli *	3 *E. coli *	1 *P *. *mirabilis *, 1 *E. coli *, 1 Other *Staph.*sp.	–	1 *E. coli* and 1 *E. faecalis *	5 *E. coli *, 3 *K *. *pneumoniae *, 1 *K *. oxytoca*,* 1 *P *. *mirabilis* 2 *s*. *aureus*, 1 *E. faecalis *
**Result**	**Sensitive**	**Intermediate**	**Resistant**	–	**Resistant**	**Resistant**

### Results of PCR for *murA, fosA, uhpT* and *glpT* genes

**Fig. 4.**
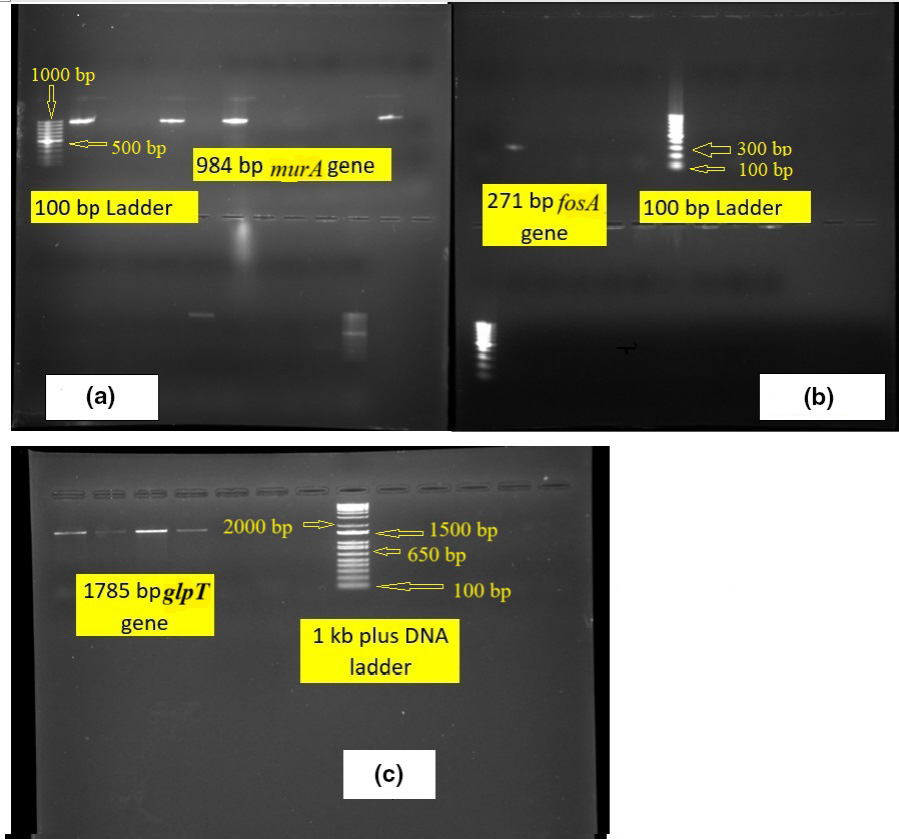
1 % agarose gel with 100 base-pair DNA ladder in Figs 4a, b and 1 kb plus DNA ladder in Fig. 4c respectively. Fig. 4a: 984 bp amplified product of *murA* gene is present at second, fifth, seventh,twelveth and nineteenth positions. Fig. 4b: 271 bp amplified product of *fosA* gene is present at second position. Fig. 4c: 1785 bp amplified product of *glpT* gene is present at first, second, third and fourth positions.

On PCR, 6 out of 21 isolates showed amplification of genes. Of these, the presence of *murA* gene was shown in four *E. coli* and one *Klebsiella pneumoniae sub sp. pneumoniae* strains, while only one isolate (*Enterococcus fecalis*) was positive for *fosA* gene (Table 5). None of the strains showed presence of *uhpT*, whereas *glpT* was detected in four *E. coli* isolates along with *murA* (Fig. 4).

**Table 5.** Fosfomycin MICs and susceptibility patterns of gene positive isolates

Fosfomycin MIC (µg ml^−1^)	128	128	128	1024	>2048	>2048
**Genes detected**	*murA, glpT*	*murA, glpT*	*murA, glpT*	*murA, glpT*	*murA*	*fosA*
**Organism**	* E. coli *	* E. coli *	* E. coli *	* E. coli *	* K. pneumoniae *	* E. faecalis *
Sensitivities*: **Ak**	S	S	S	S	S	–
**Amc**	S	R	S	S	S	–
**Cfm**	R	R	S	R	S	–
**Ctr**	S	S	S	R	R	–
**Cot**	S	S	S	R	R	–
**Nit**	S	S	S	S	S	S
**Mrp**	S	S	S	S	S	–
**Nx**	R	S	R	R	S	R
**Amp**	–	–	–	–	–	R
**HG**	–	–	–	–	–	R
**HS**	–	–	–	–	–	R
**Va**	–	–	–	–	–	S

*Abbreviations: Ak – Amikacin, Amc – Amoxiclav, Cfm – Cefixime, Ctr – Ceftriaxone, Cot – Cotrimoxazole, Nit – Nitrofurantoin, Mrp – Meropenem, Nx – Norfloxacin, Amp – Ampicillin, HG – high-level Gentamicin, HS – high-level Streptomycin, Va – Vancomycin.

## Discussion

The increasing level of antimicrobial resistance is a growing concern. The antimicrobial susceptibility patterns vary in different regions, communities, and hospitals due to the rapid evolution of resistance owing to improper use of antimicrobials [12]. Clinicians face challenge while treating UTIs caused by antibiotic-resistant uropathogens because the therapeutic choices are restricted. This is in view of increasing resistance by AmpC β-lactamases, ESBLs-producing Enterobacterales, CRE and MDR *P. aeruginosa* [19], methicillin-resistant *Staphylococci* [6], etc. The reason might be the empirical treatment given in these infections, because of the narrow spectrum of causative agents causing acute cystitis and their predictable pattern of susceptibility [20]. Moreover, the community-acquired nature of UTIs, also contributes for the increasing antimicrobial resistance among the uropathogens.

In this research study, ASB prevalence was 1173(11.4 %). This is in concordance with a study by North *et al.* in 1990, who reported 2–10 % of ASB among their study population [14]. Gayathree *et al* found the prevalence to be 6.2 %, which contrasted with this study [21].

In the present study, coliforms were responsible for majority of cases of asymptomatic bacteriuria during pregnancy, with *E. coli* (42 %) being the predominant organism. Other studies also observed *E. coli* as the major pathogen [22, 23].

Among the Gram-positive bacteria, *Enterococci* (22 %) were the main pathogen followed by other *Staph. sp.* (7.9 %) and *S. aureus* (6.4 %). In a study by Khan *et al*., the author reported *Enterococci* as the most common pathogen among the GPCs [12]. However, other studies observed *S. aureus* as the most frequent uropathogen among the Gram-positive cocci followed by other *Staph. sp.* [23].

Amoxiclav is an oral option for treatment of UTIs, but the resistance to this drug is increasing among pregnant women with UTI. In the present study, high resistance against amoxiclav was found and this fact was supported by a study in 2015 by Souza *et al*. [22]. Pais *et al*. in a study reported similar findings that urinary isolates had developed high resistance to these agents in the general population [24].

Another class of broad-spectrum antibiotics are the carbapenems, that are considered as antibiotics of choice for targeted treatment in seriously ill patients with infections of Gram-negative ESBL-producing organism. However, as there is emergence of antibiotic resistance, reduced susceptibility of some organisms to carbapenems is reported [25]. In the present study, a considerable resistance to carbapenem among Enterobacterales (40 %) and *Pseudomonas sp.* (36 %) was reported.

Among the FQs, norfloxacin exhibited high resistance (56 %) to the Enterobacterales in this study. Thakur *et al* (2013), in their study on ASB in pregnant women reported the resistance rate of 75 % to norfloxacin [26].

Among oral alternatives, nitrofurantoin and cotrimoxazole are other alternatives safe in pregnancy. However, nitrofurantoin should be avoided near term due to risk of haemolytic anaemia in G6PD deficiency patients [27].

Since, the rate of resistance among uropathogens to conventional antimicrobial agents is rising, fosfomycin is becoming popular in treating UTIs. Also, a safer option during pregnancy (category B drug). Cell-wall degradation is its mode of action, and it has been reported to be effective against *E. coli, Klebsiella sp.*, *Enterobacter sp.*, *Citrobacter sp.*, *Serratia sp.* and *E. faecalis *-related UTIs [28].

Even though fosfomycin is an old antibiotic, resistance has remained minimal, with resistance rates of 0.3–2.8% in *E. coli* and 7.2–28.6% in *Klebsiella sp.* [29]. Similarly, in our study the resistance to this drug was 3 % in *E. coli* and 3 % in *Klebsiella sp.*

Our results remained consistent by both method of testing, i.e. disc diffusion and agar dilution. However, MIC of fosfomycin may not be a strong indicator of efficacy [30], according to a study by Ballestero *et al.* in 2017. Depending on additional already existing mutations that pose no problem on the MIC determined by frequently used susceptibility testing methods, highly resistant mutants may emerge [30]. Similarly in our study *murA* genes were detected in three of the isolates having intermediate MIC of 128 µg ml^−1^.

Moreover, our study revealed the presence of only one *fosA* gene. This is in accordance with a study by Ho *et al*., where he found a low incidence of plasmid-mediated *fos* genes, in clinical *E. coli* isolates [31].

However, chromosomal resistance (*murA* and *glpT*) was more prevalent than plasmid (*fosA*) in our study. This is in concordance with a study in Europe by Castaneda-Garcia *et al.* in 2013, where the main mechanism of fosfomycin resistance was found to be chromosomal, only few isolates showed plasmid encoded gene [32].

On a deeper note, there was no work related to sequencing of genes and in near future we might use the results to detect the genetic mutations among these genes (*murA*, *glpT* and *fosA*). We would also look for other genes (*fosB*, *fosC*, *fosX*) [33] responsible for fosfomycin resistance.

## Conclusions

In conclusion, *murA* and *glpT* (chromosomal) were more commonly found genes in our study as compared to *fosA* (plasmid mediated). This was a preliminary study; so, we could not comment on the prevalence of genes on the basis of this work. Therefore, more Indian studies should be carried out in order to know the diverse distribution of genes in different parts of the country.

There was no discordance between disc diffusion results and MIC detection of strains. Other findings should be further investigated in both sensitive and resistance strains.

According to our study, the overall resistance of fosfomycin was low as compared to other oral antimicrobial agents. Therefore, it is necessary to reserve fosfomycin for management of culture confirmed multidrug-resistant infections. Better empirical drugs should be decided based on local antimicrobial susceptibility pattern of community isolates. Antimicrobial stewardship should be implemented, and strict guidelines should be adopted to stop misuse/overuse of antibiotics.

## Supplementary Data

Supplementary material 1
