# Supplementary material for: An insight into genes responsible for fosfomycin resistance among uropathogens of asymptomatic bacteriuria during pregnancy: A North Indian study
Source: Access Microbiol. 2023 Dec 11;5(12):000623.v5. doi: 10.1099/acmi.0.000623.v5 (PMC10765046; doi:10.1099/acmi.0.000623.v5)
Supplement: Supplementary material 1 [file acmi-5-623.v5-s001.pdf]

Supplementary Tables:

Table S1: **Antibiotic Susceptibility Pattern of Gram-Positive Urinary Isolates (n=438) responsible for Asymptomatic Bacteriuria during Pregnancy**

Antibiotics	*Enterococcal isolates* (n=259)	*Other Staphylococcus sp.* (n=93)	*Staphylococcus aureus* (n=75)	Total (n=438)
**Amikacin**	-	77 (83%)	61 (81%)	-
**Amoxiclav**	-	21(23%)	16 (21%)	-
**Cefoxitin**	-	22 (24%)	25 (33%)	-
**Cotrimoxazole**	-	30 (32%)	33 (44%)	-
**Fosfomycin**	257 (99%)	92 (99%)	73 (97%)	433 (99%)
**Nitrofurantoin**	199 (77%)	84 (90%)	69 (92%)	360 (82%)
**Norfloxacin**	30 (12%)	17 (18%)	17 (23%)	66 (15%)
**Vancomycin**	254 (98%)	93 (100%)	75(100)	433 (99%)
**Ampicillin**	94 (36%)	-	-	-
**Doxycycline**	38 (15%)	-	-	-
**High Level Gentamicin**	175 (68%)	-	-	-
**High Level Streptomycin**	142 (55%)	-	-	-

TableS2: **Antibiotic Susceptibility Pattern of Gram-Negative Urinary Isolates (n=775) responsible for Asymptomatic Bacteriuria during Pregnancy**

Antibiotics	*Escherichia coli* (n=495)	*Klebsiella sp.* (n=170)	*Citrobacter sp.* (n=37)	*Proteus sp.* (n=27)	*Pseudomonas sp.** (N=41)	Total (n=775 or n=734 excluding *Pseudomonas sp.*)
**Amikacin**	205 (41%)	87 (51%)	21 (56%)	10 (37%)	26 (63%)	349 (45%)
**Amoxiclav**	37 (7.4%)	5 (3%)	3 (8%)	0 (0%)	-	45 (6%)
**Cefixime**	175 (35%)	73 (43%)	13 (35%)	14 (51%)	-	275 (37%)
**Ceftriaxone**	185 (37%)	72 (42.3%)	13 (35%)	9 (33%)	-	279 (38%)
**Cotrimoxazole**	204 (41%)	77 (45.3%)	18 (48%)	12 (44%)	-	313 (43%)
**Meropenem**	297 (60%)	99 (58%)	25 (68%)	16 (59%)	26 (63%)	464 (60%)
**Fosfomycin**	484 (97%)	166 (97%)	37 (100%)	25 (93%)	-	712 (97%)
**Nitrofurantoin**	343 (69%)	77 (45.3%)	22 (59%)	-	10 (24%)	467 (60%)
**Norfloxacin**	166 (34%)	84 (49.4%)	17 (45%)	10 (37%)	-	279 (38%)
**Aztreonam***	-	-	-	-	18 (44%)	-
**Gentamicin***	-	-	-	-	28 (68%)	-
**Piperacillin tazobactam***	-	-	-	-	21 (51%)	-
**Ceftazidime***	-	-	-	-	12 (29%)	-
**Levofloxacin***	-	-	-	-	26 (63%)	-
**Cefepime***	-	-	-	-	13 (32%)	-
**Colistin***	-	-	-	-	41 (100%)	-

***-** Antibiotics used in Pseudomonas panel
